# Risk factors and predictive model for acute kidney Injury Transition to acute kidney disease in patients following partial nephrectomy

**DOI:** 10.1186/s12894-023-01325-3

**Published:** 2023-10-04

**Authors:** Sizhou Zhang, Dachun Jin, Yuanfeng Zhang, Tianhui Wang

**Affiliations:** 1 Department of Urology, People’s Hospital of Hechuan Chongqing, Chongqing, P.R. China; 2 Department of Urology, The Second Affiliated Hospital of Chongqing Medical University, Chongqing, P.R. China; 3 Department of Urology, Daping Hospital/Army Medical Center, Army Medical University, Chongqing, P.R. China; 4 Department of Urology, People’s Hospital of Fengjie, Chongqing, P.R. China

**Keywords:** Acute kidney injury, Acute kidney disease, End-stage kidney disease, Partial nephrectomy

## Abstract

**Purpose:**

Acute kidney disease (AKD) is believed to be involved in the transition from acute kidney injury (AKI) to chronic kidney disease in general populations, but little is understood about this possibility among kidney surgical populations. This study aimed to elucidate the incidence of AKD after partial nephrectomy and risk factors that promote the AKI to AKD transition.

**Methods:**

From January 2010 to January 2020, this study retrospectively collected a dataset of consecutive patients with renal masses undergoing partial nephrectomy in 4 urological centers. Cox proportional regression analyses were adopted to identify risk factors that promoted the AKI to AKD transition. To avoid overfitting, the results were then verified by logistic least absolute shrinkage and selection operator (LASSO) regression. A nomogram was then constructed and validated for AKI to AKD transition prediction.

**Results:**

AKI and AKD occurred in 228 (21.4%) and 42 (3.9%) patients among a total of 1062 patients, respectively. In patients with AKI, multivariable Cox regression analysis and LASSO regression identified that age (HR 1.078, 1.029–1.112, p < 0.001), baseline eGFR (HR 1.015, 1.001–1.030, p < 0.001), RENAL score (HR1.612, 1.067–2.437, p = 0.023), ischemia time > 30 min (HR 7.284, 2.210–23.999, p = 0.001), and intraoperative blood loss > 300ml (HR 8.641, 2.751–27.171, p < 0.001) were risk factors for AKD transition. These five risk factors were then integrated into a nomogram. The nomogram showed excellent discrimination, calibration, and clinical net benefit ability.

**Conclusion:**

Around 3.9% patients following partial nephrectomy would transit from AKI to AKD. Intraoperative blood loss and ischemia time need to be diminished to avoid on-going functional decline. Our nomogram can accurately predict the transition from AKI to AKD.

**Supplementary Information:**

The online version contains supplementary material available at 10.1186/s12894-023-01325-3.

## Introduction

Partial nephrectomy (PN) should be adopted for renal masses whenever possible to maximally protect renal function [1]. Nevertheless, acute kidney injury (AKI) is observed in approximately 1/5 of patients after surgery [2]. Approximately 8-22% of patients eventually progress to chronic kidney disease (CKD) one year after surgery, and this incidence gradually increases over time [2–6].

Both AKI and CKD have been well-defined to improve research efforts and refine subsequent management strategies and recommendations [7]. However, patients with renal abnormalities in function and/or structures who might not fulfill the criteria of AKI (< 7days of renal function abnormality) or CKD (> 90days of renal function abnormality) are not well-studied. Traditionally, AKI and CKD are regarded as two separate entities, representing two different courses of diseases. AKI refers to a condition in which rapid renal dysfunction occurs, while CKD represents a slowly progressing course of chronic kidney damage. However, AKI and CKD are increasingly recognized as related entities and they may represent different stages of the same pathophysiological process [8, 9]. In this regard, acute kidney disease (AKD) is defined to describe the continuum from AKI to CKD in general populations among whom these pathophysiological processes are ongoing by Kidney Disease Improving Global Outcomes (KDIGO) guidelines [9, 10]. A growing body of evidences noted that AKD should be detected, evaluated, and treated before patients transited to CKD or even end-stage kidney disease (ESKD) [7, 11].

Similar to AKI, AKD is divided into four stages based on serum creatinine levels 7–90 days after the first insult [9, 10]. For medical patients, this insult process is usually bilateral and systemic, representing a declining renal function process. In kidney surgery patients, the subsequent decline in renal function after the operation is normally unilateral (loss of kidney units, ischemia time and a healthy contralateral kidney). In addition, patients who undergo PN have fewer comorbidities than medical patients. It seems plausible that these differences might lead to different long-term outcomes. AKI after PN is negatively correlated with long-term renal function and mortality [12]. Risk factors need to be awarded and avoided so as to early break the AKI-AKD-CKD-ESKD chain. In this regard, the early prediction of transition from AKI to AKD after PN has important clinical implications.

Thus, this study aimed to elucidate the incidence of AKD after PN, identify the risk factors that promote AKI transiting to AKD, and construct a risk model to predict the transition from AKI to AKD in renal surgery patients.

## Materials and methods

### Patient selection

Our study protocol was approved by the institutional review board of the Institutional Review Board of the Second Affiliated Hospital of Chongqing Medical University Ethical Committee and registered in the Chinese Clinical Trial Registry (Registration No.: ChiCTR2000034080 (23/06/2020)). Informed consent was waived due to its retrospective design in the training cohorts. And Informed consent was obtained in the validation cohorts. We routinely produced PN with main-artery clamping and warm ischemia technique.

**Fig. 1:**
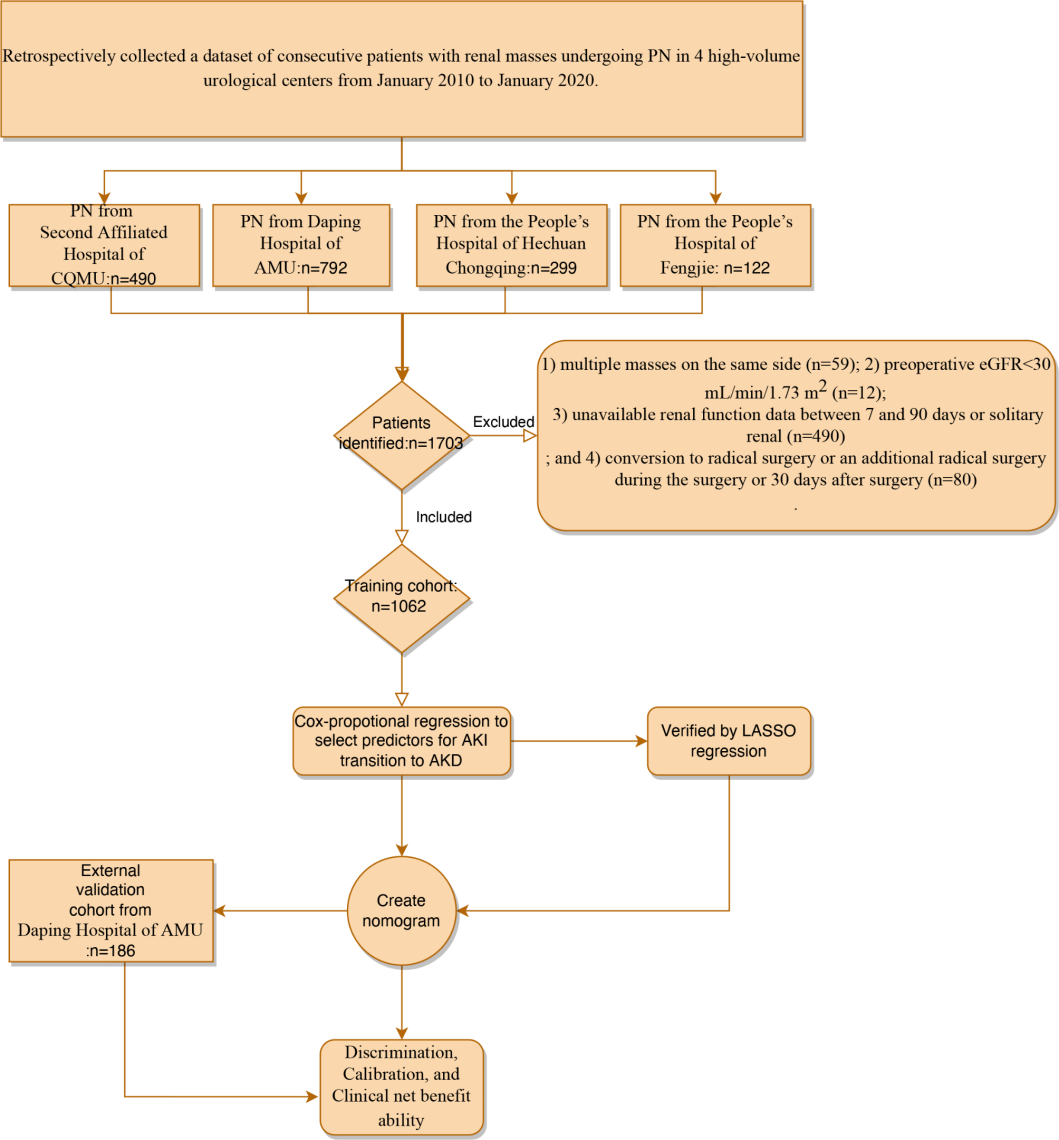
The workflow and patient selection process. PN: partial nephrectomy

Figure 1 shows the workflow and patient selection process. Patients with single-site ipsilateral renal lesions who underwent PN from January 2010 to January 2020 were retrospectively reviewed from 4 urology centers (The Second Affiliated Hospital of CQMU, The People’s Hospital of Hechuan Chongqing, Daping Hospital of AMU, and People’s Hospital of Fengjie). Patients conforming to any one of the following criteria were excluded from this study: (1) multiple masses on the same side (n = 59); (2) preoperative estimated glomerular filtration rate (eGFR) less than 30 mL/min/1.73 m^2^ (n = 12); (3) solitary kidney(n = 28); 4)unavailable renal function data between 7 and 90 days (462); and (4) conversion to radical surgery or an additional radical surgery during the surgery or 30 days after surgery (n = 80). A prospective cohort with the same criteria (from Daping Hospital of AMU during 2020.01-2021.06) served as validation (n = 186). All patients were typically followed up every 1–3 months for the first 1 year and then every 6–12 months per year, including eGFR and renal imaging (sonograph or CT scan).

### Data collection and definitions

Demographic characteristics, surgical outcomes and kidney functional results during follow-up were collected from the electronic databases in 4 centers (The Second Affiliated Hospital of CQMU, The People’s Hospital of Hechuan Chongqing, Daping Hospital of AMU, and People’s Hospital of Fengjie) and filled into a predesigned standardized sheet. AKI was defined according to the KDIGO serum creatinine criteria as increase in serum creatinine by ≥ 0.3 mg/dL (≥ 26.5 micromol/L) within 48 h, or increase in serum creatinine to ≥ 1.5 times baseline, which is known or presumed to have occurred within the prior seven days, or urine volume < 0.5 mL/kg/hour for six hours [13]. AKD was defined as an elevation in serum creatinine to at least 1.5-fold from baseline between 7 and 90 days according to ADQI 16 workgroup [9]. Stage 1 includes patients for whom serum creatinine levels are higher than 1.5 times baseline but within 2.0 times baseline levels. Stage 2 includes patients for whom serum creatinine levels are higher than 2.0 times baseline but within 3.0 times baseline levels. Stage 3 includes patients for whom serum creatinine levels are higher than 3.0 times baseline or require temporary RRT. Stage 4 includes patients for whom require ongoing RRT. The primary endpoint was the transition from AKI to AKD. The secondary endpoint was the development to ESKD during the follow-up.

### Statistical analysis

Data are expressed as the mean ± standard deviation or medians (interquartile ranges) for continuous variables and as the percent frequency for categorical variables. During the comparison study between patients with and without AKI, continuous variables were compared using Student’s t test or the Wilcoxon rank sum test, while categorical variables were compared using the chi-square test between the two groups. Univariable and multivariable Cox regression analyses were adopted to identify risk factors for AKD. The results were then verified by logistic least absolute shrinkage and selection operator (LASSO) regression, a machine learning method, to avoid overfitting and underpowering. Kaplan–Meier analysis was used to analyze relationships between AKD transition and ESKD during the long-term follow-up. A nomogram was created to visualize the prediction. The constructed nomogram was then assessed by discrimination (concordance index [C-index]), calibration (the observed incidence versus the nomogram-predicted probability by the locally weighted scatterplot smoothing [LOWESS] method) and decision curve analysis. STATA version 16.1 software (Stata Corp., College Station, TX., USA) was used for the data analysis. A difference of p < 0.05 indicated statistical significance and the level of precision was present according to EUA guideline [14].

## Results

AKI and AKD occurred among 228 (21.5%) and 42 (3.9%) patients, respectively. Patients with AKD were identified as older (57.7 ± 13.5 vs. AKI group: 54.2 ± 11.7), with a higher eGFR (131.2 ± 42.8 ml/min/1.73 m^2 vs. the AKI group: 120.8 ± 30.9 ml/min/1.73 m^2^), with a higher rate of ischemia time over 30 min (26.2% vs. 14.9%), and had more intraoperative blood loss (210 ± 103ml vs. 160 ± 92ml). The demographic information and surgical outcomes are presented in Table 1. All baseline variables were comparable between the training and validation cohorts except intraoperative blood loss.

**Table 1:** Demographic information and surgical outcomes of 1062 consecutive patients who underwent open, laparoscopic or robot-assisted PN from 4 urology centers

Variables	Training cohort					Validation cohort
	All patients (n = 1062)	Non-AKI (n = 834)	AKI (n = 228)	AKD(n = 42)	p value	n = 186
Patient-related factors						
Age (years old)	53.9 ± 14.5	53.9 ± 15.2	54.2 ± 11.7	57.7 ± 13.5	0.8	55.4 ± 13.9
Male (%)	667(62.8)	529 (63.4)	168(73.7)	29(69.0)	0.003*	105(56.5)
Body mass index	25.9 ± 3.1	25.8 ± 3.1	26.2 ± 3.3	26.4 ± 4.2	0.09	26.0 ± 3.2
Right lesion (%)	285(44.7)	245(44.6)	40(45.5)	23(54.7)	< 0.001*	84(45.2)
Baseline eGFR(ml/min1.73 m^2)	116.7 ± 32.2	115.6 ± 32.5	120.8 ± 30.9	131.2 ± 42.8	0.031*	120 ± 30.1
Diabetes	199(18.7)	153(18.3)	46(20.2)	11(26.1)	0.6	37(19.9)
Hypertension	297(28.0)	227(27.2)	69(30.2)	15(35.7)	0.4	60(32.3)
ASA classification						
1–2 (%)	739(69.6)	578(69.3)	161(70.6)	27(64.3)	0.7	140(75.3)
3–4 (%)	323(30.4)	256(30.7)	67(29.4)	15(35.7)		46(24.7)
Tumor-related information						
Tumor diameter (cm)	3.9 ± 1.5	3.8 ± 1.4	4.0 ± 1.8	4.2 ± 1.7	0.07	3.7 ± 1.6
RNS	6.0(IQR5-7)	5.8(IQR5-7)	6.1(IQR5-7)	6.8(IQR6-8)	< 0.001*	6.0(IQR5-7)
4–5	290(27.3)	253(30.3)	37(16.2)	5(11.9)		59(31.7)
6–8	741(69.7)	571(68.4)	170(74.6)	28(66.7)		121(65.1)
≥ 9	31(2.9)	10(1.2)	21(9.2)	9(21.4)		6(3.2)
Pathological information						
Malignancy (%)	934(87.9)	725(86.9)	209(91.7)	38(90.4)	0.001*	171(91.9)
Benign (%)	128(12.1)	109(12.0)	19(8.3)	4(9.6)		15(8.1)
T stage						
T1a	624(58.8)	502(60.2)	122(53.5)	24(57.1)	0.037*	105(56.4)
T1b	284(26.7)	222(26.6)	62(27.2)	12(28.6)		65(35.0)
T2a	70(6.6)	46(5.5)	24(10.5)	6(14.3)		16(8.6)
Unknown	84(7.9)	64(7.6)	20(8.7)	0		0
Surgical type						
Open (%)	289(27.2)	217(26.0)	72(31.5)	15(35.7)	0.1	42(22.6)
Laparoscopic (%)	595(56.0)	469(56.2)	126(55.2)	21(50.0)		109(58.6)
Robot-assisted (%)	178(16.8)	148(17.8)	30(13.1)	6(14.3)		35(18.8)
Surgery-related outcomes						
Warm ischemia time ≥ 30 min (%)	115(10.8)	81(9.7)	34(14.9)	11(26.2)	0.030*	18(9.7)
Surgery time (min)	131 ± 46	130 ± 50	138 ± 25	146 ± 40	0.020*	135 ± 43
Length of hospital stay (day)	6.0 ± 2.1	5.9 ± 2.0	6.5 ± 2.3	6.7 ± 2.5	< 0.001*	6.1 ± 1.9
Transfusion (%)	42(3.9)	31(3.7)	11(4.8)	3(7.1)	0.4	4(2.2)
Intraoperative blood loss (ml)*	147 ± 75	140 ± 70	160 ± 92	210 ± 103	< 0.001*	135 ± 52
Postoperative complications	146(14.0)	101(12.1)	45(19.7)	11(26.2)	0.005*	21(11.3)
Clavien I (%)	49(4.7)	40(4.7)	9(3.9)	1(2.3)		5(2.7)
Clavien II (%)	61(5.8)	40(4.7)	21(9.2)	4(9.5)		14(7.5)
Clavien III (%)	34(3.2)	26(3.1)	8(3.5)	2(4.8)		3(1.6)
Clavien IV (%)	12(1.2)	5(0.6)	7(3.1)	4(9.5)		1(0.5)
AKI stage 1 (%)	203(19.5)		203(89.1)			32(16.1)
AKI stage 2/3 (%)	25(2.4)		25(10.9)			6(3.2)
AKD stage 1 (%)	32(3.1)			32(76.2)		7(70.0)
AKD stage 2/3 (%)	10(1.0)			10(23.8)		3(30.0)
Progression to CKD or CKD up-stage	242(22.8)	172(20.6)	70(30.7)	31(73.8)	0.020*	37(19.9)

PN: partial nephrectomy; ASA: American Society of Anaesthesiologists; IQR: interquartile range; AKI: acute kidney injury; AKD: acute kidney disease; RNS: RENAL nephrometry score. Comparison between the training cohort and validation cohort: all p > 0.05 except intraoperative blood loss. *: p < 0.05

**Fig. 2:**
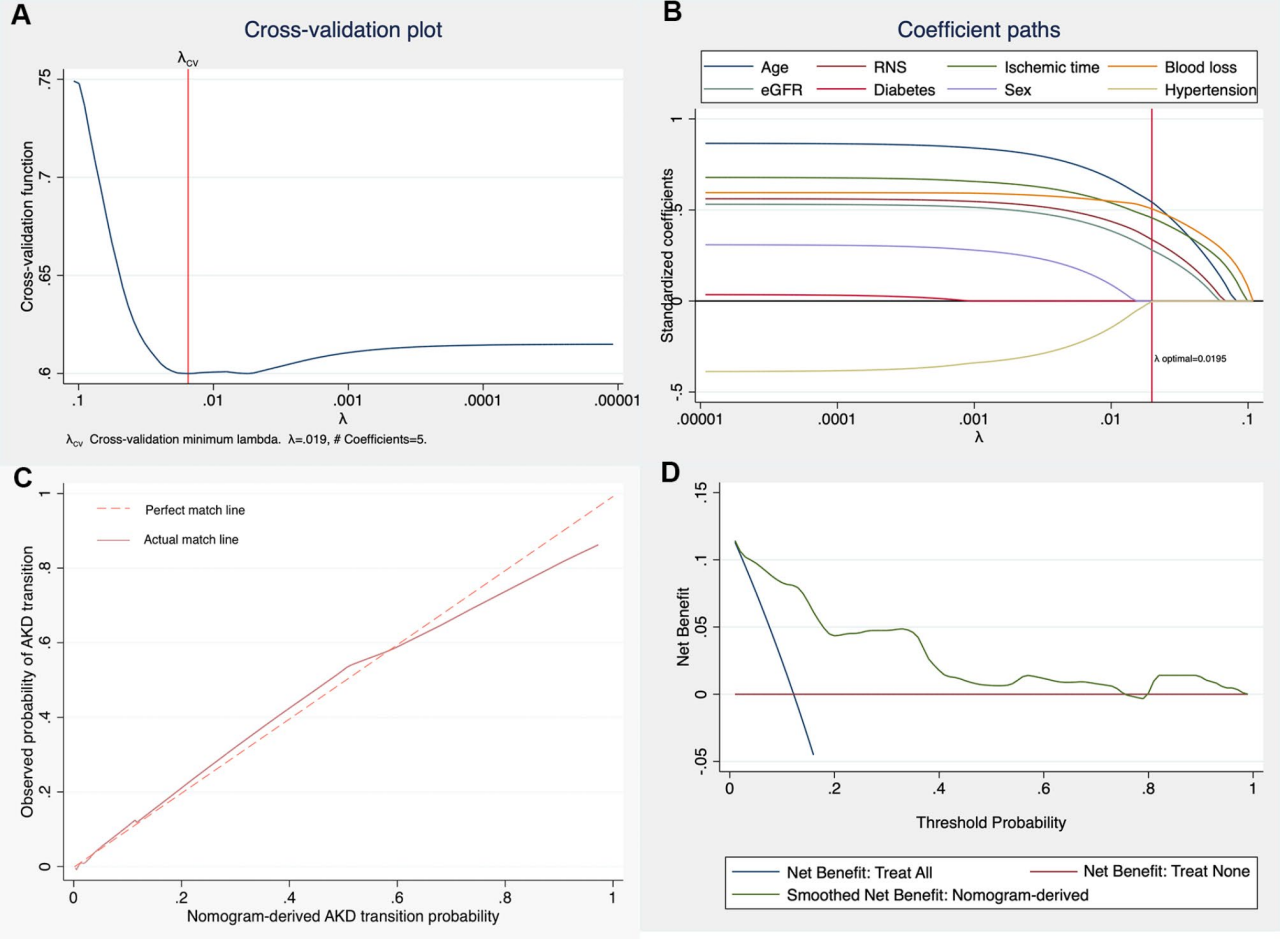
**2A.** Cross-validation plot identified the optimal value at λ = 0.019. **2B**. Risk factor selection using the logistic least absolute shrinkage and selection operator (LASSO) regression. The LASSO standardized coefficient profiles of the nonzero variables of the predictors. A coefficient path plot was produced against the ln(lambda) sequence. Reading from left to right, as the penalty factor (λ) increases, the standardized coefficients decrease and finally reach zero, which means exclusion from the model. At the optimal value (λ = 0.019), age, preoperative eGFR, ischemia time, intraoperative blood loss and RNS remained in the model. LASSO: logistic least absolute shrinkage and selection operator. RNS: RENAL Nephrometry Score. **2C**. The calibration curve for the predictive nomogram exhibited a high agreement between the actual probability and predicted probability of the AKD transition. **2D**. Decision curve analyses demonstrating the net benefit associated with the use of the nomogram-derived probability for the prediction of the transition possibility from AKI to AKD.

**Fig. 3:**
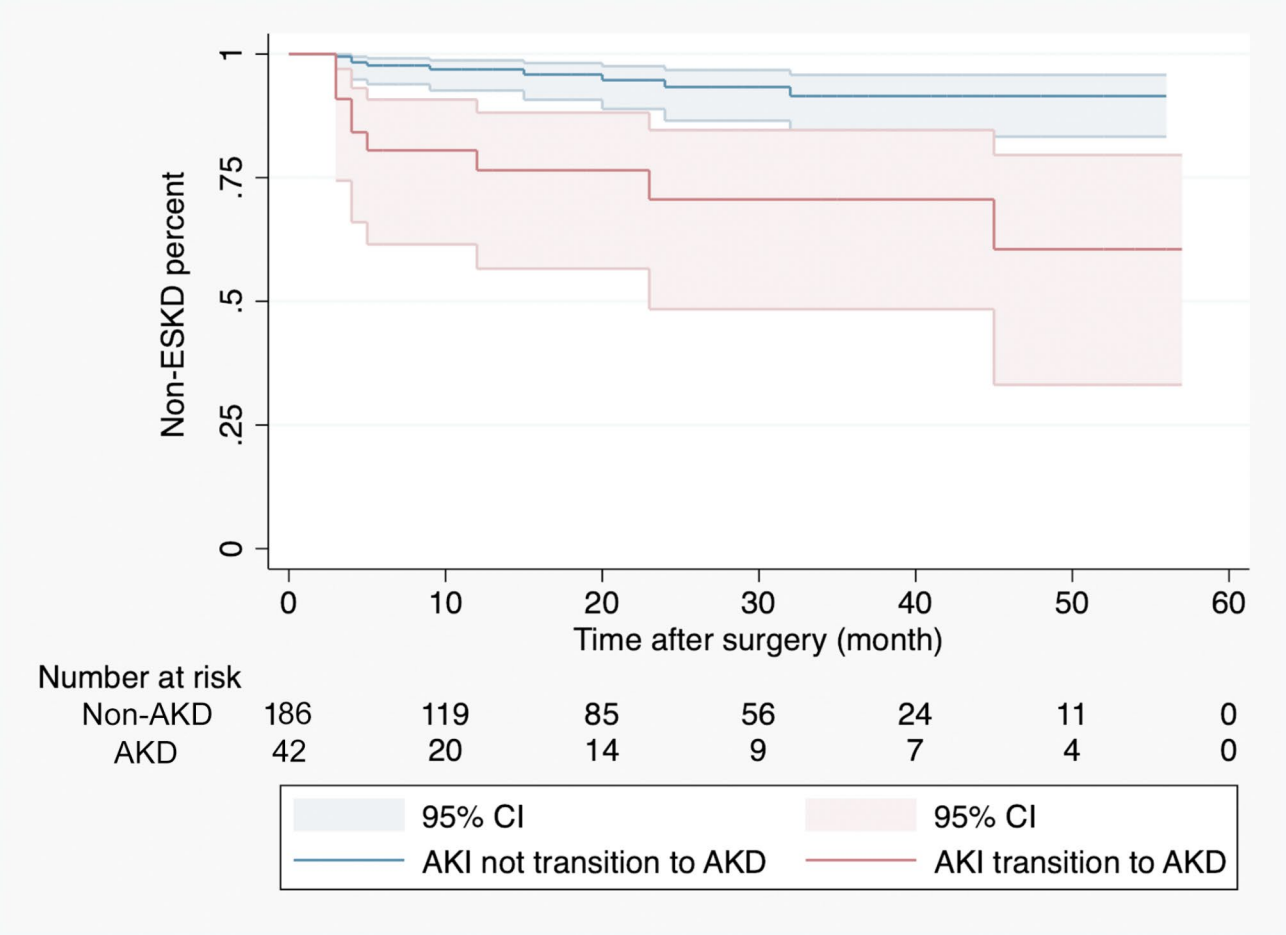
Kaplan–Meier plot comparing the transiting or not transiting from AKI to AKD groups that developed ESKD. ESKD: end-stage kidney disease

After the PH (Schönfeld test) test, univariable and multivariable Cox regression analyses identified that age (HR 1.078, 1.029–1.112, p < 0.001), baseline eGFR (HR 1.015, 1.001–1.030, p < 0.001), RENAL score (HR1.612, 1.067–2.437, p = 0.023), ischemia time > 30 min (HR 7.284, 2.210–23.999, p = 0.001), and intraoperative blood loss > 300ml (HR8.641, 2.751–27.171, p < 0.001) were risk factors for AKD transition (Table 2). Specifically, the relationship between intraoperative blood loss and AKD emerged as nonlinear, with a plateau from 0 to around 300 mL of intraoperative blood loss and an increase afterward. To avoid overfitting, we ran a cross-validation test (10-fold) to choose the optimal penalty factor (λ = 0.019) (Fig. 2A). In the logistic LASSO regression analysis, it was observed that as the penalty factor (λ) increased to the optimal value of 0.019, preoperative eGFR, tumor diameter, ischemia time and blood loss remained in the model, indicating that this result was reliable (Fig. 2B). These results are consistent with the previous multivariable Cox regression analysis. During the follow-up of an average of 19 months (3–60 months), a total of 18 patients developed ESKD. Patients transiting from AKI to AKD was more likely to developing to ESKD compared with patients not transiting to AKD (Log-rank test: p < 0.001). (Fig. 3).

**Table 2:** Univariable and multivariable cox-proportional regression analysis of influence factors for transition from AKI to AKD.

	Univariate cox regression				Multivariate cox regression			
	HR	95% CI		p	HR	95% CI		p
Age	1.072	1.031	1.114	< 0.001*	1.078	1.029	1.129	< 0.001*
Preoperative eGFR	1.017	1.005	1.029	0.005*	1.01	1.001	1.030	0.034*
Diabetes								
No	1.000							
Yes	1.206	0.782	2.164	0.4				
Hypertension								
No	1.000							
Yes	0.508	0.228	1.129	0.10				
Ischemia time								
< 30 min	1.000				1.000			
≥ 30 min	6.679	2.604	17.128	< 0.001*	7.284	2.210	23.999	0.001*
RNS	1.671	1.198	2.331	0.002	1.612	1.067	2.437	0.023*
Intraoperative blood loss								
< 300ml	1.000				1.000			
> 300ml	4.757	1.706	13.263	0.003*	8.641	2.751	27.171	< 0.001*
p value from Schönfeld test								0.2

h: Hazard ratio; CI: confidential interval; eGFR: estimated glomerular filtration rate; RNS: RENAL nephrometry score

**Fig. 4:**
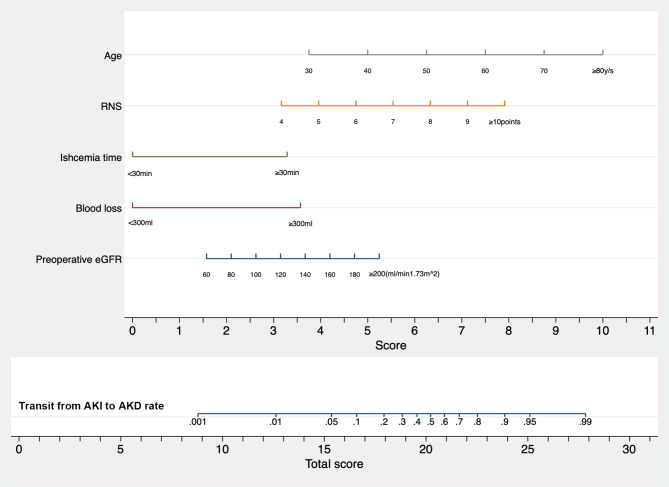
A nomogram predicting the transition possibility from AKI to AKD. Instructions: to estimate a patient’s probability, locate the patient’s value on each variable axis. Draw a vertical line from that value to the top *Score* scale for determining how many points are assigned by that variable value. Then, the points from each variable value are summed. Locate the sum on the *Total score* scale and vertically project it onto the bottom axis, thus obtaining a final risk probability

A nomogram was then created and validated based on risk factors to visualize presentation (Fig. 4). The predictive ability as measured by concordance-index was 0.891(95% CI 0.830,0.953). The calibration curve for the predictive nomogram exhibited a high agreement between the actual probability and predicted probability of the AKD transition (Fig. 2C). Finally, we utilized decision curve analysis (DCA) to determine the clinical utilities of this predictive nomogram, which then demonstrated that the nomogram was clinically useful (Fig. 2D).

A prospective cohort (a total of 186 cases, 10 cases of AKD) with the same criteria (from 2020.01 to 2021.06) was served as validation. The predictive ability was 0.903(95% CI 0.842,0.964). Calibration plot and DCA also showed excellent outcomes. (Supplementary materials 1.)

## Discussion

In this study, we explored the incidence of AKD and elucidated its role in PN surgical population. We further made a practical nomogram to accurately predict the transition from AKI to AKD which could help to break the AKI-AKD-CKD-ESKD chain in an early stage.

AKD was first proposed by the KDIGO AKI workgroup to define any acute condition that impacts kidney function, eGFR < 60 ml/min/1.73 m2, a decrease in GFR by > 35%, an increase in serum creatinine of > 50%, or any kidney damage lasting < 3 months in 2012. In 2017, the ADQI 16 Workgroup described AKD as acute or subacute damage for a duration of between 7 and 90 days after exposure to an AKI initiating event and added a staging system [9]. In 2021, the KDIGO workgroup propose a broader term of “kidney diseases and disorders” (KD) to describe abnormalities of kidney function and/or structure, where AKI was descripted as a subset of AKD [7]. However, for patients with AKI or AKD, the risk of long-term functional decline or ESKD were highly different (Fig. 3). In order to differentiate AKD from AKI and stratify different risk groups, we adopted 2017 AKD as our main standard.

AKD is defined as a bridge from AKI to CKD in the general population [7, 9]. For patients who undergo PN, this gap between AKI and CKD has not yet been fully discussed. Previous studies only focused on the relationship among AKI, CKD and long-term renal function [3, 12, 15, 16]. More recently, AKI progression to AKD has attracted increasing attention, as it may provide more insights into functional preservation. It is understandable that even for the urological community, the presence of AKI after kidney surgery is often seen as a transitory functional failure [17]. For most patients who experience AKI, their renal function eventually recovers, and their decreased function remains stable after an initial decrease [18–20]. In the study with the longest follow-up time, Bertolo et al. [19] found that with a minimum follow-up of over 5 years, renal function following PN remained stable after 1 year. Our data suggested that patients who progressed to AKD from AKI were much more likely to experience persistent functional deterioration, and this process can continue for much longer during the follow-up. For this reason, these patients require more attention with a longer close follow-up time.

In the literature, it is inevitable that 9-37% of patients experience AKI after PN [17], and in our study, approximately 18.4% of these AKI patients later developed AKD after AKI. In a study enrolling 16,968 critically ill patients, this number could be as high as 40% [21] or 45% in a hospitalized population [22]. Our data were much lower than in the medical population, as surgical patients may have fewer comorbidities and a higher functional reserve, and the parenchymal loss itself also “overestimates” the actual rate [2, 9, 23]. More recently, Hu et al. demonstrated that approximately 28% of patients had AKD in partial or radical nephrectomy populations, but in their study, the exact rate was unknown in PN population [24], which is consistent with our results. Moreover, it has also been proven that in non-kidney surgical patients, AKD is associated with adverse renal functional outcomes [23, 25].

The presence of AKD might be the continuum of non-recovery or progressing AKI in two dimensions: severity and duration, and these two dimensions are often parallel. Both AKI severity and its duration have been identified as risk factors for adverse long-term renal function [2, 3, 12, 19, 21]. On the other hand, patients who develop AKD may have lower preoperative renal reserves and more comorbidities [23, 25]. These long-lasting medical conditions can still have a fundamental influence on postoperative functional recovery. In addition, patients with AKD are more fragile when a second insult occurs after kidney surgery. These three aspects may explain its high rate of progressing to functional deterioration.

The risk factors for AKI and CKD after PN have been well studied [3, 17–20]. In our study, unsurprisingly, AKD shared almost the same risk factors as AKI and CKD, which was in line with other studies [23]. Interestingly, intraoperative blood loss was identified as risk factors even adjusted after other well-known variables. Our findings are in line with a recent study by Rosiello et al. [26], which revealed that intraoperative bleeding over 500ml is a risk factor for the development of CKD. This might ascribe to the high complexity of tumor, diminished renal perfusion, and high chance of blood transfusion injury. Ischemia time and intraoperative blood loss are only two modifiable risk factors and surgeons should lower the adverse impact of these two as possibly as one can (i.e. high risk patients requires more experienced hands, use clampless or sutureless technique to diminish or eliminate ischemia time) [27].

Unlike nonsurgical medical patients, parenchymal resection, ischemia time, and blood loss were three different risk factors, and a healthy contralateral kidney might also mitigate all of these risks [18]. There was concern that AKI might be overestimated considering the loss of kidney parenchyma [28]. The influence of this one-time insult (PN surgery: ischemia damage and parenchymal loss) gradually diminishes over time and becomes insignificant [19]. Under this condition, it seems reasonable to adopt AKD as a more reliable indicator of a risk of long-term functional deterioration. This progressing renal dysfunction course, namely, AKI-AKD-CKD-ESKD, initiated by surgery, might be the consequence of multiple factors, ranging from pre-existing damage, long-lasting comorbidities, ischemia time, and parenchymal loss affecting the contralateral kidney condition. Considering the adverse events associated with low functional reserve, it is crucial to create a better risk stratification system, as current systems have only focused on preoperative factors [15, 16]. AKD may be included to establish a more comprehensive predictive model in the future.

To the best of our knowledge, this is one of the very few studies focused on the incidence of AKD and its role in long-term renal function after PN. Our sample size was large, and the follow-up duration was relatively long. Several of our findings could be noteworthy. First, the overall incidence of AKD in the surgical population is low compared with the hospitalized population, whilst nearly 1/5 of AKI patients after PN transit to AKD. Second, AKD is an interim period from AKI to CKD after PN, which is consistent with nonsurgical medical populations. Third, modifiable risk factors, like intraoperative blood loss, should be avoided to achieve better function results. Fourth, AKD is strongly associated with long-term renal functional deterioration, and thus, patients progressing to AKD from AKI require more attention to avoid continuous functional decline, and a multidisciplinary joint effort might be needed.

The main limitation of our study is its retrospective nature and multicenter design. However, it is difficult to complete such a study in a single center due to the low incidence of AKD. Despite our cohort number was large, the AKD number is limited also due to its low incidence. In addition, some patients may be missed in terms of renal outcomes 7–90 days after surgery, and the available patients might have more severe functional damage, which in turn might overestimate the AKD incidence. Tumor characteristics (i.e., the actual volume of the loss of renal parenchyma) and the surgical experience were not collected from our database. In addition, potential risk factors (i.e., diabetes, hypertension control status, proteinuria) were not accessible during the follow-up. Proteinuria was proved to be strongly correlated with postoperative AKI, functional decline [29].

## Conclusions

In conclusion, our study found that the overall incidence of AKD in the surgical population is low. Modifiable risk factors (intraoperative blood loss, ischemia time) should be diminished as possibly as a surgeon can to avoid on-going functional decline as they were the only modifiable risk factors for AKD, i.e. open surgery or surgeons with more expertise should be considered for complex renal mass to diminish ischemia time and blood loss. A close follow-up for patients with high-stage AKD should be implemented, and possible interventions should be evaluated in future studies to break the AKI-AKD-CKD course to prevent long-term renal functional deterioration after PN.

### Electronic supplementary material

Below is the link to the electronic supplementary material.

Supplementary Material 1

## Data Availability

The raw data used to support the findings of this study are available from the corresponding author upon reasonable request.
