# Supplementary material for: Risk factors and predictive model for acute kidney Injury Transition to acute kidney disease in patients following partial nephrectomy
Source: BMC Urol. 2023 Oct 4;23:156. doi: 10.1186/s12894-023-01325-3 (PMC10552238; doi:10.1186/s12894-023-01325-3)
Supplement: Supplementary file 1 — Supplementary Material 1 [file 12894_2023_1325_MOESM1_ESM.pdf]

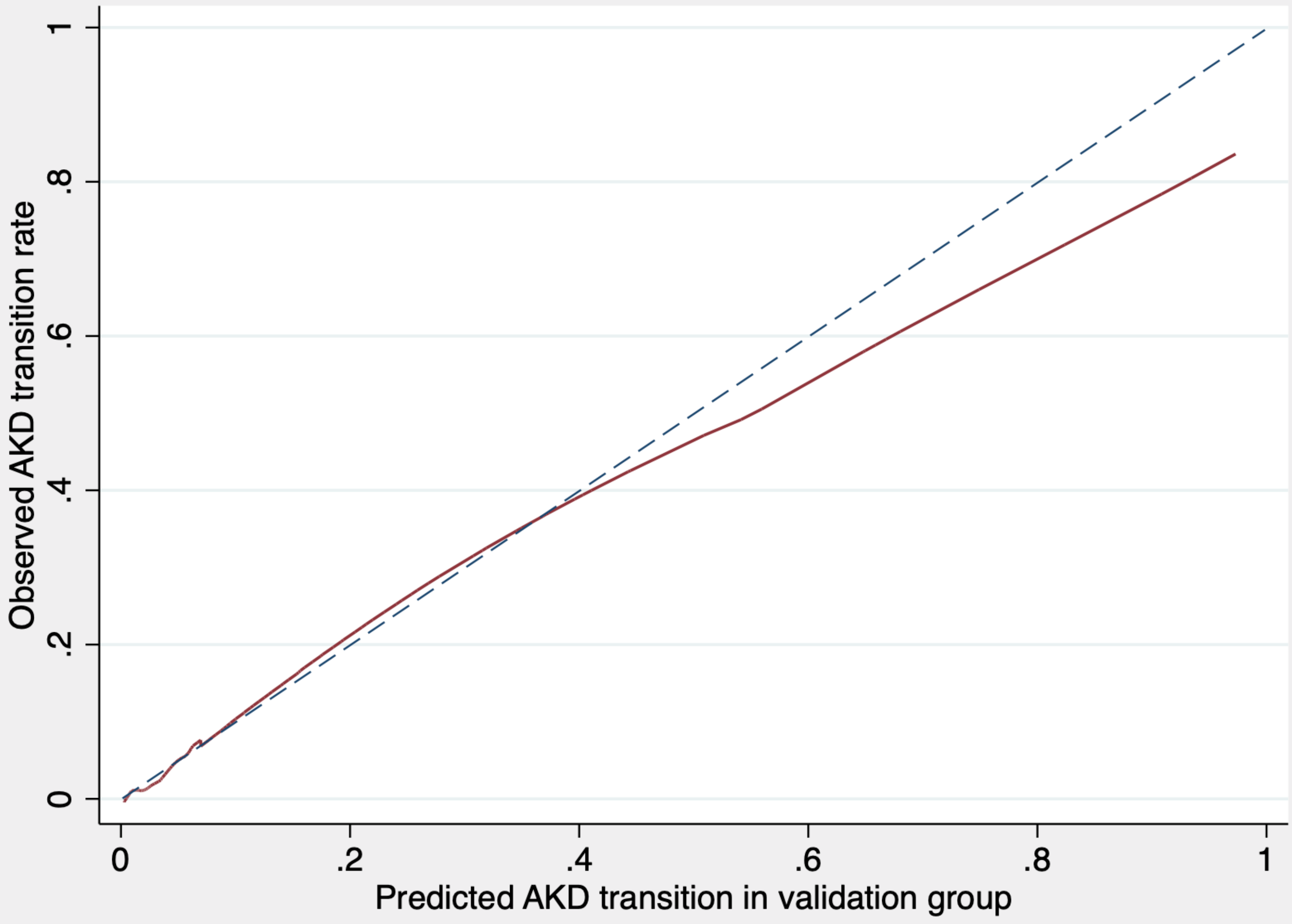

Observed AKD transition rate
0
.2
.4
.6
.8
1
0
.2
.4
.6
.8
1
Predicted AKD transition in validation group

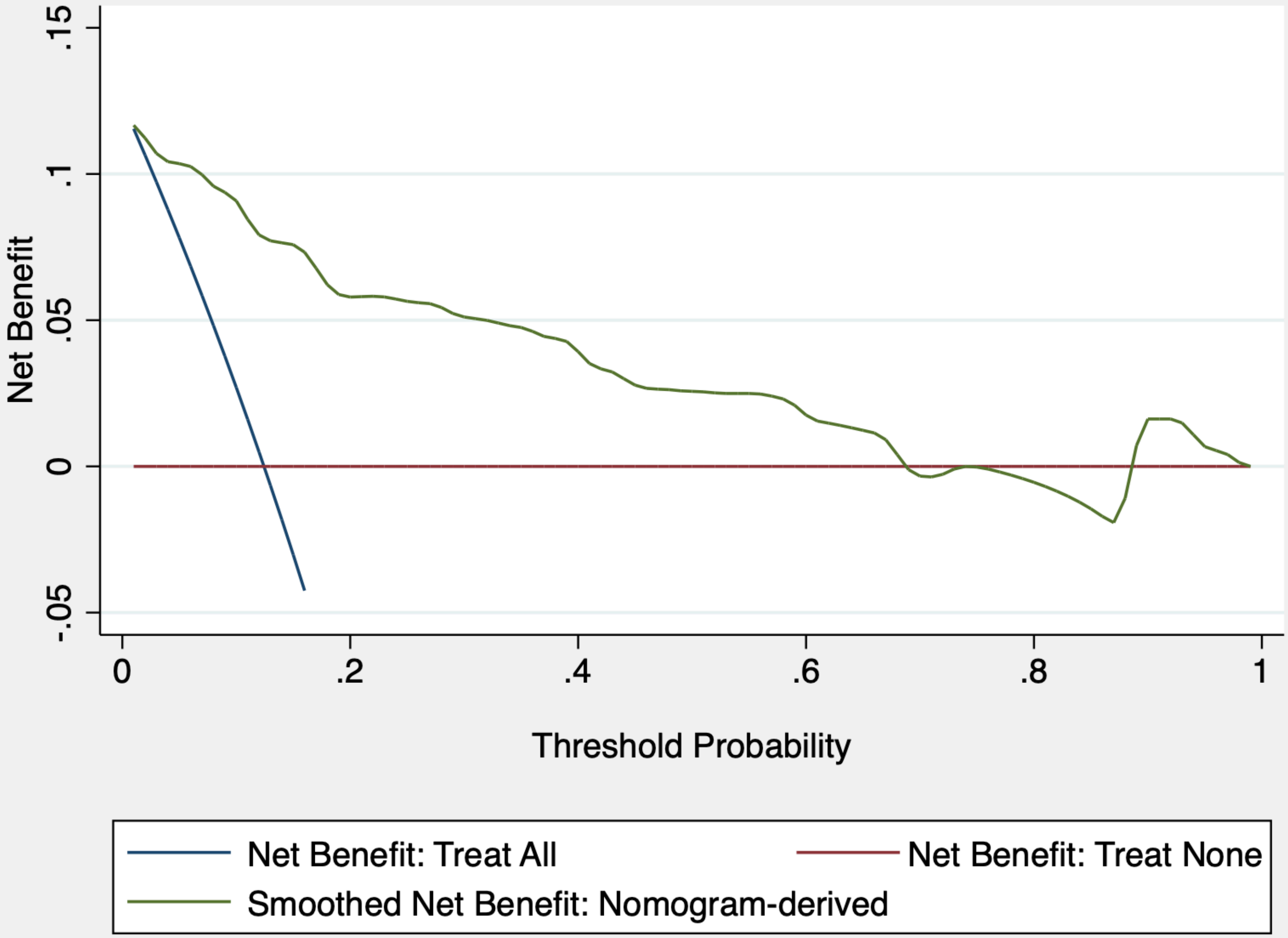
Net Benefit
.15
.1
.05
0
-.05
0
.2
.4
.6
.8
1
Threshold Probability
Net Benefit: Treat All
Net Benefit: Treat None
Smoothed Net Benefit: Nomogram-derived

Supplementary Figure 1. Calibration plot of observed (actual) vs. nomogram-predicted probability of the transition from AKI to AKD in the external cohorts.

Supplementary Figure 2. Decision curve analyses demonstrating the net benefit associated with the use of the nomogram-derived probability for the transition from AKI to AKD in the external cohorts.
